# Composted Sewage Sludge Influences the Microbiome and Persistence of Human Pathogens in Soil

**DOI:** 10.3390/microorganisms8071020

**Published:** 2020-07-09

**Authors:** Nikola Major, Jasper Schierstaedt, Sven Jechalke, Joseph Nesme, Smiljana Goreta Ban, Marko Černe, Søren J. Sørensen, Dean Ban, Adam Schikora

**Affiliations:** 1 Institute of Agriculture and Tourism, Karla Huguesa 8, 52440 Poreč, Croatia; smilja@iptpo.hr (S.G.B.); marko@iptpo.hr (M.Č.); dean@iptpo.hr (D.B.); 2 Leibniz Institute of Vegetable and Ornamental Crops, Department Plant-Microbe Systems, Theodor-Echtermeyer-Weg 1, 14979 Großbeeren, Germany; jasper.schierstaedt@julius-kuehn.de; 3 Institute for Phytopathology, Centre for BioSystems, Land Use and Nutrition, Justus Liebig University Giessen, Heinrich-Buff-Ring 26, 35392 Giessen, Germany; sven.jechalke@julius-kuehn.de; 4 Section of Microbiology, University of Copenhagen, Universitetsparken 15, 2100 Copenhagen, Denmark; joseph.nesme@bio.ku.dk (J.N.); sjs@bio.ku.dk (S.J.S.); 5 Julius Kühn-Institut, Federal Research Centre for Cultivated Plants, Institute for Epidemiology and Pathogen Diagnostics, Messeweg 11/12, 38104 Braunschweig, Germany

**Keywords:** soil microbiome, sewage sludge compost, *Salmonella enterica*, internalization, *Brassica rapa*

## Abstract

Composted sewage sludge (CSS) gained attention as a potential fertilizer in agriculture. Application of CSS increases soil microbial activity and microbial biomass, however, it can also lead to increased chemical and microbiological risks. In this study, we performed microcosm experiments to assess how CSS reshapes the microbial community of diluvial sand (DS) soil. Further, we assessed the potential of CSS to increase the persistence of human pathogens in DS soil and the colonization of Chinese cabbage (*Brassica rapa* L. subsp. *pekinensis* (Lour.) Hanelt). The results revealed that CSS substantially altered the prokaryotic community composition. Moreover, addition of CSS increased the persistence of *Salmonella enterica* serovar Typhimurium strain 14028s and *S.* *enterica* serovar Senftenberg in DS soil. However, the enhanced persistence in soil had no impact on the colonization rate of *B.* *rapa* grown on soil inoculated with *Salmonella*. We detected *Salmonella* in leaves of 1.9% to 3.6% of plants. Addition of CSS had no impact on the plant colonization rate. The use of sewage sludge composts is an interesting option. However, safety measures should be applied in order to avoid contamination of crop plants by human pathogens.

## 1. Introduction

Many processes in the soil ecosystem are governed by microorganisms. Prominent examples include promotion of plant growth, nutrient cycling and degradation of pollutants [1]. These processes are threatened by climate change, changed precipitation patterns and soil degradation due to inappropriate land management practices [2]. Even though taxonomic and functional diversity of the plant microbiome are progressively characterized [3], up to now, not many soil microorganisms have been cultivated in a laboratory environment and therefore their functions are largely unknown [2,4]. There is a close interaction between the microbial community and the plant host. This interaction is important for plant health and also for the final structure of the microbial community associated with the host plant [5]. Previous studies revealed that soil is the main microbiological reservoir shaping the community structure in the rhizosphere, and root exudates are the main drivers for the occurring change [6]. Furthermore, by altering the rhizobiome the plant can promote a suppressive soil environment in which the proliferation of a pathogen is inhibited [5]. This may be of great importance since both plant and human pathogenic bacteria can contaminate crop plants and cause yield losses or disease outbreaks [7,8,9].

The use of sewage sludge as fertilizer may contribute to sustainable agriculture and nutrient recycling. The application of sewage sludge increases soil microbial activity and microbial biomass [10] but could also lead to increased chemical and microbiological risks [11]. Human pathogens potentially preset in sludge might contaminate soil-grown fresh produce, which might pose a risk for the consumer [12]. European legislation concerning the use of sewage sludge in agriculture demands specific treatments before its application [13]. Sewage sludge, in addition to pathogenic microorganisms, may contain other organic and inorganic pollutants [14]. Therefore, a sludge stabilization procedure is used in order to remove its adverse characteristics such as odor and putrescence, as well as pathogenic bacteria. There are several methods for sewage sludge stabilization which includes lime stabilization and anaerobic or aerobic digestion. Sludge composting as an aerobic stabilization method has been proven a reliable and low-cost strategy for sludge treatment prior to agricultural use [15,16].

*Brassicaceae* are consumed traditionally in all parts of the world, especially in Asia, Europe and North America [17,18,19]. They are rich in vitamins, phenolic compounds and glucosinolates and have many health benefits including anticancerogenic, antimicrobial and antidiabetic effects [20,21,22]. Growing in the field, plants are constantly challenged by environmental factors including pathogens [12,23]. Irrigation water, manure, livestock and wildlife are the usual sources of pathogens [24]. In addition, diverse post-harvest, handling and distribution-related procedures to which produce are exposed pose a high risk of contamination by microorganisms [25].

An important aspect of food contamination is the internalization of pathogens in plant tissues. Internalized pathogens are protected from surface washing and may withstand the usual cleaning procedures. The biology and ecology of pathogen-produce interactions gained particular interest after several disease outbreaks connected to fresh produce [26]. The major routes for pathogen internalization include natural plant openings like stomata, lenticels, lateral root emergence sites, etc., or sites with physical or biological damage. Moreover, irrigation or washing water is often involved in internalization of microorganisms in plant tissues [27].

Unfortunately, the soil environment harbors human pathogens. They may be either part of the indigenous microbial community, for example *Listeria monocytogenes*, or can persist in soil for long periods, for example *Salmonella enterica*. The ability to persist in soils seems to be governed by the diversity of the native soil microbial community. In the case of *S. enterica*, our previous study revealed a significantly lower persistence in soil with a natural diversity compared to soil with an experimentally reduced microbial diversity [28]. At the same time, it was shown that several biotic and abiotic factors, such as soil type and fertilizer applied, potentially influence the internalization of human pathogens in plant tissues [29].

The aim of this work is to assess the impact of sewage sludge compost amendment on the structure of microbial community and the persistence of human pathogens in soil environment. To the best knowledge of the authors, the interaction between the soil microbial communities, composted sewage sludge (CSS) application and internalization of human pathogenic bacteria into plant tissues has not been investigated before. We therefore assessed the impact of CSS on (a) the soil microbiome, (b) the persistence of *S. enterica* in soil and (c) the colonization rate of *Brassica rapa* (Chinese cabbage) phyllosphere by two *S. enterica* strains.

## 2. Materials and Methods

### 2.1. Bacterial Strains

*Salmonella enterica* serovar Typhimurium strain 14028s was obtained from Dr. Isabelle Virlogeux-Payant (INRA Tours, France). *S. enterica* serovar Senftenberg, was provided by Professor John Coia (Scottish Reference Centre for Salmonella, Glasgow, Scotland, UK) and Dr. Nicola Holden (James Hutton Institute, Dundee Scotland, UK). Spontaneously rifampicin-resistant mutants were isolated by inoculation of an overnight culture on LB agar plates containing rifampicin (50 mg/L). Resistant colonies were plated onto LB agar with rifampicin to confirm resistance.

### 2.2. Sewage Sludge Composting

Domestic dewatered aerobically-stabilized sewage sludge obtained in larger quantities from a local wastewater treatment plant (WWTP), (Istria, Croatia) was used to produce compost. The study was designed within the experimental farm of the Institute of Agriculture and Tourism, Croatia. The composting experiment started on 1. June 2017 in a sheltered concrete enclosure (77 × 77 × 77 cm). Composting biomass was prepared by mixing of raw sewage sludge and wheat straw at a dose of 40 kg of straw per one m^3^ of sludge. Temperature in the center of the compost pile was monitored daily using temperature smart sensors (HOBO, ONSET, Bourne, MA, USA) connected to USB micro station data logger (HOBO, ONSET, Bourne, MA, USA). The aeration was enabled according to standard measures for compost production [30]. The moisture level of the compost was maintained within 55% to 60% and controlled according to EPA [31]. After 3 months of composting, the compost’s maturity was evaluated according to Canet et al. [30].

### 2.3. Microcosm Experiment

For the microcosm experiments, a sandy soil from Großbeeren (52° 21′ N, 13° 18′ E, Germany) was used. It was characterized as Arenic-Luvisol [32], with less silty sand and 5.5% clay [diluvial sand (DS)] which was sieved (2 mm) and adjusted to 50% of its water holding capacity resulting in a final water content of 112 mL per one kg of soil [33]. The soil was amended with CSS at a final concentration of 25.71 g per kg dry soil according to the Nitrates Directive [34]. *S.* Typhimurium 14028s and *S.* Senftenberg were grown at 37 °C overnight in LB supplemented with rifampicin (50 mg/L). Bacterial cells were pelleted (1500 × *g*, 10 min) and washed twice with 10 mM MgCl_2_. Soil was mixed with bacterial solution to obtain a final concentration of 10^6^ colony forming units (CFU) per g dry soil. Soil was filled into polystyrene flowerpots (11 cm diameter, 9 cm height) with approximately 400 g soil per pot, and in each pot two Chinese cabbage plants (*Brassica rapa* L. subsp. *pekinensis* (Lour.) Hanelt, PREDURO F1, Takii Europe B.V., De Kwakel, The Netherlands) at the three- to four-leaf stage were transplanted.

The experiment was designed with two possible scenarios of *S. enterica* soil contamination: (a) via fertilizer where *S. enterica* was introduced into soil together with the CSS (“stored” treatment) 28 days prior to *B. rapa* transplanting (mixing day), and (b) via irrigation water where *S. enterica* was introduced in soil with water (“fresh” treatment) on the day *B. rapa* plants were transplanted (day 0, planting day). For mock-inoculation (control) 10 mM MgCl_2_ was used. Pots were placed individually on plates with fleece and incubated for 56 days at 20 °C in the greenhouse. Plants were watered as needed. Additional mineral fertilizer was added with the irrigation water from day 37 on. Soil was sampled at seven timepoints including mixing day (day –28), planting day (day 0), days 7, 14, 21, 35 and 56 after transplanting. Before sampling, soil in each pot was mixed thoroughly. Plant leaves were sampled prior to soil sampling on the same day.

### 2.4. DNA Extraction, 16S rRNA Gene Amplification and Amplicon Sequencing

Total DNA was extracted from four independent biological replicates (four independent pots) of 0.5 g soil using the FastDNA SPIN Kit for Soil (MP Biomedicals, Heidelberg, Germany) and purified with GENECLEAN SPIN Kit (MP Biomedicals, Heidelberg, Germany) according to the manufacturer’s instructions. The approximately 460 bp fragment covered hypervariable regions V3–V4 and was originally published by Yu et al. [35] and modified as described in Sundberg et al. [36]. In a second PCR reaction step, the primers additionally included Illumina specific sequencing adapters and a unique dual index combination for each sample. Small PCR products (<100 bp) were removed using HighPrep PCR paramagnetic beads (MagBio Genomics, Gaithersburg, MD, USA) according to the manufacturer’s instructions, using a 0.65:1 of beads:PCR reaction volume ratio. Samples were pooled and adjusted to equimolar concentrations using SequalPrep Normalization Plate (96) Kit (Invitrogen, Carlbad, CA, USA) and pooled using a 5 µL volume for each sample. The pooled samples library was concentrated using DNA Clean and Concentrator™-5 kit (Zymo Research, USA) and sequenced using 2 × 250 bp paired-end high-throughput sequencing using Illumina MiSeq Reagent Kits version 2 on an Illumina MiSeq platform (Illumina, USA). Sequencing was performed both on “stored” and “fresh” treatments with *S.* Typhimurium 14028s on planting day (day 0) and day 35 post planting, as well as on soil before inoculation (mixing day, day –28) and control (MgCl_2_-inoculated) soil.

### 2.5. Sequence Analysis

Raw sequences were trimmed of primer sequences using cutadapt [37]. After merging, the sequences were clustered with 97% pairwise sequence similarity using UPRASE [38] and chimeras were removed using UCHIME [39]. Taxonomic annotation was performed using mothur “classify.seqs” and “method = wang” with a minimum bootstrap probability of 0.8 [40] against RDP database trainset 16 [41]. After sequencing a total of 1,412,486 reads, a total of 13,799 OTUs were obtained with an average number of 32,848 reads per sample. A single sample with less than 1,000 reads was removed. After removing OTUs with low abundance (less than four counts) and low variance (less than 10%) 1,844 OTUs remained for downstream analysis. Alpha-diversity was calculated at the OTU level using the Shannon Index. Significant differences between treatments were calculated using one-way analysis of variance (ANOVA) or *t*-test. Beta-diversity at the OTU level, indicating similarities of the microbial community composition between different treatments, was analyzed using the Jensen–Shannon divergence method with non-metric multidimensional scaling (NMDS) prior to testing significant differences with analysis of similarities (ANOSIM). Differences in abundance between treatments at the phylum level were calculated using Student’s *t*-test with Benjamini–Hochberg adjusted *p* values. The microbial community differential abundance between treatments was statistically analyzed with a generalized linear model fit and a likelihood ratio test post-hoc false discovery rate multiple correction test using the “edgeR” package in R 3.6.3 [42]. Univariate statistical analyses were performed using the Statistica software package version 13.4 (Tibco Software, Inc.). Species richness, NMDS and ANOSIM were calculated with the MicrobiomeAnalystR package [43]. Sequences were uploaded to NCBI with the ID number PRJNA630375.

### 2.6. Detection of Salmonella enterica in Soil and Plant Tissues

In order to determine the colony forming units (CFU) of *Salmonella enterica* in soil, from each of the four biological replicates two technical replicates of serial dilutions for each soil sample were dropped twice on XLD agar (Merck KGaA, Darmstadt, Germany), supplemented with rifampicin (50 mg/L). CFU counts were enumerated after incubation at 37 °C for 20 h. In order to quantify the colonization rate of Chinese cabbage phyllosphere, samples were taken 7, 14, 21, 35 and 56 days after transplantation into diluvial sand (DS) soil. Only leaves without contact to soil were harvested. Leaves were chopped with sterile scissors and incubated in 10 mL Buffered Peptone Water (BPW) for 20 h at 37 °C. Subsequently, 10 µL of enrichments were dropped on XLD agar (Merck KGaA, Darmstadt, Germany) supplemented with rifampicin (50 mg/L). After incubation at 37 °C for 20 h, the presence of *S. enterica* was indicated by black color of bacterial colonies. Additionally, 190 µL of the *Salmonella*-selective Rappaport-Vassiliadis Broth was inoculated with 10 µL of the BPW enrichment. After incubation at 42 °C for 20 h, samples were dropped on XLD agar supplemented with rifampicin (50 mg/L). After incubation at 37 °C for 20 h, samples positive for *Salmonella* were enumerated.

The decrease rates of *Salmonella* CFU counts between soil treatments were compared by linear modeling using the aov function conducted in R [42], where a significant interaction between the factors time (day) and treatment (“fresh” or “stored”) indicated different *Salmonella* persistence. Due to the imbalanced design (day –28 only for the “stored” treatment), only days 0 to 56 were included in the analysis.

## 3. Results

### 3.1. Composition of Prokaryotic Community Changes upon Time, Sludge Compost Amendment and S. enterica Presence

**Figure 1:**
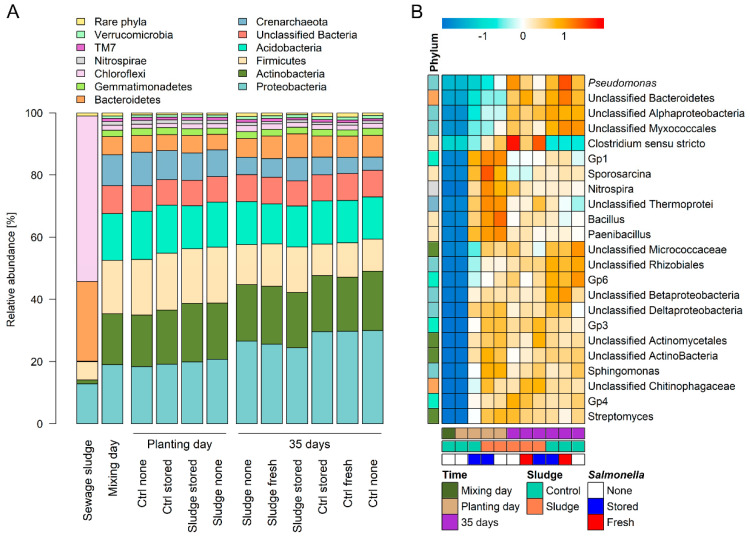
Taxonomic distribution of microbial phyla and treatment responding groups. Diluvial sand soil was treated with composted sewage sludge (CSS) and inoculated with *Salmonella enterica* serovar Typhimurium strain 14028s (*Salmonella*). Total DNA was sampled after mixing the soil with CSS, directly after planting *B. rapa* and 35 days after planting. The relative abundance was calculated as percentage of 16S rRNA gene sequences belonging to a particular phylum in each sample and the mean is displayed for each group in a stacked bar plot (**A**). Taxonomic groups, which differ in their relative abundance between treatments, were identified using negative binomial regression, generalized linear model fit and a likelihood ratio test post-hoc false discovery rate multiple correction test (*p* < 0.05). These groups are represented using a color code (center-scaled mean of read counts out of four replicates) (**B**). For a complete list see Supplementary Table S2. Each experimental variant was represented by at least three replicates.

The composition of the soil prokaryotic community was analyzed on three different timepoints (day –28, 0 and 35) using the 16S rRNA gene fragment amplicon sequencing. Taxonomic analysis of the prokaryotic community revealed that the soil communities were composed mostly of 12 bacterial phyla. In all tested experimental variants, regardless of the time, presence of composted sewage sludge (CSS) or *Salmonella*, the most abundant were: Proteobacteria, Actinobacteria, Firmicutes, Acidobacteria, Crenarchaeota, Bacteroidetes, Gemmatimonadetes, Chloroflexi, Nitrospirae, TM7 and Verrucomicrobia (Figure 1A).

The analysis of relative abundance between the phyla indicated that the three major factors assessed here (time, CSS and *Salmonella enterica*) had different impact on the microbial composition. Proteobacteria, Actinobacteria, Bacteriodetes, Chloroflexi and Verrucomicrobia significantly increased in relative abundance, while Firmicutes, Acidobacteria, Crenarchaeota and Nitrospirae decreased (Table 1) 35 days after transplanting the *Brassica rapa* (Chinese cabbage) plants. The addition of the CSS to the soil significantly decreased the relative abundance of Acidobacteria while the relative abundance of Chloroflexi increased (Table 1). The presence of *S. enterica* had no impact on the composition of the soil prokaryotic community on phylum level (Table 1). Furthermore, two proteobacteria, an unclassified genus of order Rhizobiales and an unclassified order of the class Betaproteobacteria, were more abundant in samples without CSS as well as the genus GP6 of Acidobacteria. In contrast, only one genus, *Paenibacillus*, which belongs to the phylum Firmicutes, was more abundant in samples with CSS (Figure 1B).

**Figure 2:**
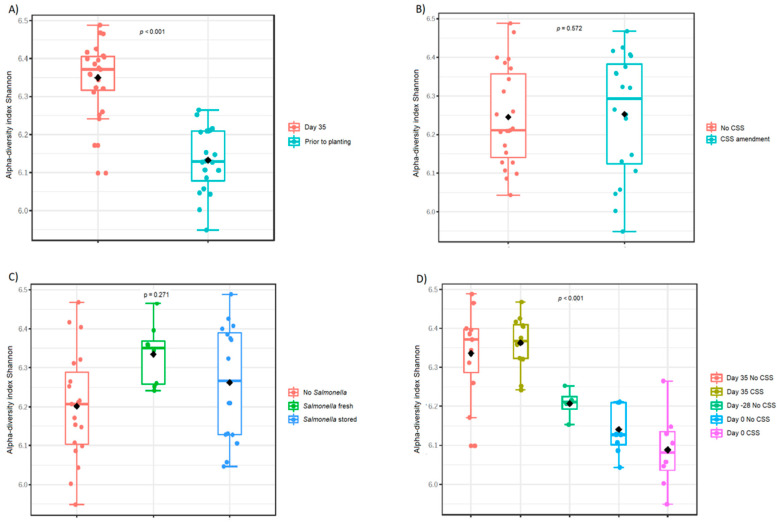
Diversity in the prokaryotic community represented using the Shannon index. The effects of *B. rapa* growth (**A**), amendment with sewage sludge compost (**B**), absence or presence of *Salmonella* (**C**), and time after planting (**D**) were calculated. The significance was tested using one-way ANOVA. The boxes represent the interquartile range between the first and the third quartile and the lines mark the median. In total, 43 samples were analyzed.

The influence of time and the presence of the CSS and *Salmonella* on the prokaryotic alpha diversity in the soil samples was analyzed in the following steps. The Shannon index of the soil prokaryotic community, representative of alpha diversity, increased 35 days after the planting of *B. rapa* (Figure 2A,D). The addition of CSS did not change the alpha diversity of the soil communities neither did the presence of *S. enterica* (Figure 2B,C).

**Figure 3:**
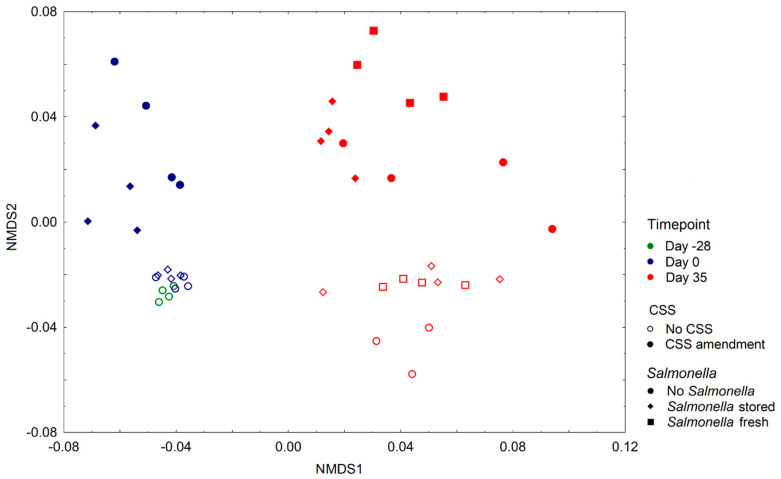
Beta diversity between the prokaryotic communities in diluvial sand (DS) soil. The non-metric multidimensional scaling (NMDS) analysis of the microbial community and analysis of similarities (ANOSIM) were performed according to the sampling timepoint (green: mixing day, blue: planting day, red: 35 days after planting). [ANOSIM] R: 0.85035; *p*-value < 0.001, [NMDS] stress = 0.096213, sludge amendment (empty: without sludge, solid color: sludge amended) [ANOSIM]. R: 0.36323; *p*-value < 0.001, [NMDS] stress = 0.096213 and *Salmonella enterica* presence (circles: no *Salmonella enterica*, diamonds: *Salmonella enterica* introduced via fertilizer, squares: *Salmonella enterica* introduced via irrigation) [ANOSIM]. R: 0.095536; *p*-value < 0.036, [NMDS] stress = 0.096213. *p*-values ≤ 0.05 were considered significant.

In the next step we analyzed the beta-diversity between the samples. The non-metric multidimensional scaling (NMDS) analysis paired with ANOSIM revealed significant differences in samples after 35 days of *B. rapa* growth and to a lesser extent after sludge compost addition to the soil (Figure 3). The presence of *S. enterica* in soil had no impact on the prokaryotic beta-diversity if CSS was not added. Moreover, significant differences between the “stored” and “fresh” treatments were observed when the soil was amended with CSS. The NMDS plot indicated that there was no difference in the community profile of control DS soil during the first 28 days of incubation prior to planting (mixing day vs planting day without sludge), while there was a noticeable change in the prokaryotic community after the addition of CSS during the same period (mixing day vs planting day with sludge). After 35 days of the plants growing, the soil prokaryotic community changed noticeably in both the control and sludge amended samples (Figure 3). Different taxa of the phyla Firmicutes, Crenarchaeota, Actinobacteria, Acidobacteria, Proteobacteria, Bacteriodetes and Nitrospirae were significantly influenced by time (Figure 1 and Table 1.). The correlation analysis revealed a positive correlation between Firmicutes, Crenarchaeota, Nitrospirae and Acidobacteria (Supplementary Table S1). Additionally, Acidobacteria correlated positively with Gemmatimonadetes. On the other hand, a positive correlation was observed between Proteobacteria, Bacteroidetes and Verrucomicrobia. The two groups were negatively correlated (Supplementary Table S1). Time seems to play the key function, while the presence of CSS plays only a minor role and inoculation with a *Salmonella* strain seems to have no impact on the number of most abundant groups.

### 3.2. Amendment with Composted Sewage Sludge Increased the Persistence of Salmonella in Soil

**Figure 4:**
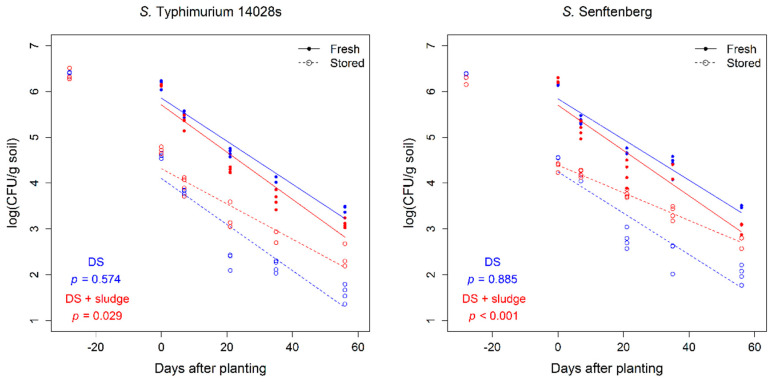
Colony forming unit (CFU) counts and persistence dynamics of *Salmonella enterica* in DS soil. A decrease in CFU counts of *S*. Typhimurium 14028s and *S*. Senftenberg per g dry soil with or without sewage sludge compost amendment are shown. All experimental variants were performed in quadruplicates and CFU counts were assessed by dropping duplicates of duplicate serial dilutions on XLD plates supplemented with rifampicin (50 mg/L). The dynamics of *Salmonella* persistence in DS soil according to entry route, soil treatment and serovar are expressed as linear regressions. *Salmonella* was applied either 28 days before planting (“stored”) together with the sludge (DS + sludge) or without sludge (DS) or immediately before planting on day 0 (“fresh”). The decrease rates between fresh and stored treatments were compared for soil amended with and without sludge by linear modeling and *p* values below 0.05 indicate a significant difference. Due to the imbalanced design (day –28 only for the “stored” treatment) only days 0 until 56 were included in the modeling.

In the next step, we wondered if the amendment with sludge would influence the persistence of human pathogens in soil. To this end, DS soil either with or without CSS was inoculated with *Salmonella enterica* serovar Typhimurium strain 14028s (*S*. Typhimurium 14028s) or *Salmonella enterica* serovar Senftenberg (*S.* Senftenberg) and their persistence was monitored throughout the experiment. When introduced to DS soil together with CSS 28 days prior to transplanting, a better persistence (lower decrease rates) of both *S.* Typhimurium 14028s and *S.* Senftenberg was observed compared to the persistence of *Salmonella* applied on planting day to already fertilized soil (Figure 4). On the other hand, no differences were observed between decrease rates of *Salmonella* applied a month prior to planting and *Salmonella* applied on planting day to unfertilized soil (Figure 4).

### 3.3. Amendment with Composted Sewage Sludge Has No Impact on Colonization of B. rapa by Salmonella

The sludge’s impact on *Salmonella* persistence in soil motivated us to assess if this would have an impact on the plant colonization rate if crop plants grow on contaminated soil(s). The relocation of *S.* Senftenberg and *S*. Typhimurium 14028s from soil to the upper plant tissues (phyllosphere) was therefore compared between *B. rapa* plants grown on soil with or without the sewage sludge compost amendment. Leaf samples were taken over 56 days after planting, which corresponds to the usual vegetation period for Chinese cabbage. From a total of 678 *B. rapa* plants that grew on soils inoculated with *S. enterica,* 1.9% to 3.8% tested positive for the presence of *Salmonella* in their phyllosphere (Table 2). ANOVA revealed that the presence of CSS or the *S. enterica* serovar had no detectable impact on the colonization rate (Table 2). However, a three-fold increase (*p* = 0.066) in the colonization rate was observed when *S. enterica* was introduced to the soil together with the plant (simulating contamination via irrigation water) if compared to the scenario in which *Salmonella* was introduced together with the CSS amendment (Table 2). The number of positive *S. enterica* detections in the *B. rapa* phyllosphere increased with time and at day 21 we observed the highest number of colonized plants. In subsequent timepoints, no positive tests for *S. enterica* were observed (Table 2).

## 4. Discussion

When nutrients are limited, in order to survive the plant may attract beneficial bacteria to the rhizosphere by sharing sugars and other secondary metabolites and, consequently, increase the selective pressure on the soil microbiome [44]. On the other hand, the plant is less prone to divert sugars and metabolites if enough nutrients are available [45].

In this study, we investigated how the prokaryotic community changes during cultivation of Chinese cabbage in soil with or without CSS. During 35 days of the plants’ growth, Proteobacteria, Actinobacteria, Acidobacteria, Bacteroidetes, Chloroflexi and Verrucomicrobia increased in their relative abundance while a significant decrease was observed for Firmicutes, Acidobacteria, Crenarchaeota and Nitrospirae. The addition of CSS significantly boosted the relative abundance of Firmicutes and Chloroflexi and lowered the relative abundance of Acidobacteria. Proteobacteria displayed the biggest increase, rising from 20.1% to 30.5%, on average. Proteobacteria are important in carbon, nitrogen and sulfur cycling [46]. Different taxa which were enriched in CSS-treated soil declined in abundance over time (Figure 1). Some, such as Nitrospirae and *Sporosarcina*, are known to play a role in the biogeochemical nitrogen cycle (i.e., ammonia oxidation and denitrification). Interestingly the genus *Pseudomonas*, a known denitrifier, was enriched in abundance at later timepoints. These results indicate a shift in the taxonomic distribution after CSS treatment while the biological functions are maintained. Actinobacteria are mainly studied as sources of bioactive compounds and their role in their natural soil habitat is still greatly unexplored [47]. It is known that Actinobacteria may employ both direct and indirect mechanisms to influence plant growth and protection. Direct mechanisms involve the production of vital factors for plant growth such as growth hormones and the assistive action on nitrogen fixation, phosphate solubilization or iron acquisition [48]. Bacteroidetes were reported to decrease in abundance with the intensification of agricultural use [49] while Chloroflexi increased in relative abundance after organic fertilization [50], possibly due to an increase in C sinking function [51]. Firmicutes encompass known plant pathogenic bacteria [52] and although the introduction of the plant lowered their relative abundance, the addition of CSS significantly increased the relative abundance of this phylum. Sewage sludge is rich in Firmicutes, especially Bacilli and Clostridia, and the composting process further enriches their abundance [53]. Acidobacteria are mainly present in soils and their influence on the ecosystem is not well understood [54]. The relative abundance of this phylum decreased with the introduction of the plant, and their presence is further decreased with the introduction of CSS. They are versatile heterotrophs and possess genetic traits that suggest they are best suited to low-nutrient environments [55]. Although Nitrospirae benefits plants by oxidizing nitrite to nitrate [56], we observed a decrease in relative abundance after 35 days of Chinese cabbage growth.

The effect of organic amendments on *Salmonella* persistence in soils is still not clear. There are several published studies on the persistence of various *Salmonella enterica* serovars in soils with organic amendments with opposite outcomes. Jechalke et al. [29] reported a prolonged persistence of *Salmonella* in soils amended with poultry and pig slurry based organic fertilizers. On the other hand, a study on the influence of sewage sludge amendment on the persistence of *S.* Typhimurium LT2 in soil reported decreased CFU numbers in soils amended with sewage sludge compared to control soils [57]. The investigated parameters in our study focused on the introduction of *Salmonella* together with the organic amendment or directly before planting simulating contamination through irrigation water. The obtained results indicate that the differences between *Salmonella* persistence in the two scenarios could possibly arise from the incubation period. In the scenario where *Salmonella* was introduced at the same timepoint as CSS, it had an equal incubation time as other competitive microbes and likely higher concentrations of available nutrients. In this scenario, we observe better *Salmonella* survival rates compared to control samples in which *Salmonella* was added to soil 28 days after fertilization. The results obtained in this study could be explained by the recent report by Schierstaedt et al. [28], where it was demonstrated that the abundance of *Salmonella enterica* decreased faster in soils with a highly diverse prokaryotic community compared to soils with low diversity, underlying the importance of the native soil microbiome in suppressing pathogenic bacteria. Shah et al. [58] investigated the persistence of *S.* Newport in soils with or without heat-treated chicken manure pellet amendment. The authors reported a significantly higher survival rate of *Salmonella* in soils with the organic amendment, reaching 91 dpi versus 35 dpi for control soils. Our study showed that the presence of *Salmonella* had no impact on the relative abundance of the soil microbiome on phylum level, which is in line with a previous study, which showed that *Salmonella* influenced the soil microbiome only when the prokaryotic diversity was very low [28].

The total ratio of *Salmonella*-colonized plants (phyllosphere) was between 1.9% and 3.6%. Although we have observed different *S. enterica* persistence after the addition of CSS, this had no detectable impact on plant colonization. The same outcome was observed for both tested *S. enterica* serovars. However, plant colonization seemed higher (*p* = 0.066) when *S. enterica* was introduced together with the plant, via the irrigation water. Additionally, the highest colonization rate was observed 21 days after planting. Coincidentally, in subsequent sampling points no *S. enterica* was detected in the *B. rapa* phyllosphere, despite the presence of *Salmonella* in the soil. Confocal laser scanning microscope (CLSM) analysis did not reveal potential mechanisms of *Salmonella* root colonization and transport to upper plant tissue (data not shown). Previously, Jechalke et al. [29] investigated the colonization rate of *S. enterica* on corn salad and lettuce leaves through root internalization. Similar to this report, the authors reported 0.5% to 0.9% of plants colonized with *Salmonella*. The internalization of human pathogenic bacteria into edible parts poses a significant risk for consumers. Koukkidis et al. [59] reported that traces of juices released from salad leaves after being damaged could enhance *S. enterica* proliferation even under refrigerated conditions. Moreover, the endogenous leaf microbiota was largely unresponsive to such leaf juice, suggesting that *Salmonella* could gain an advantage, using fluids released by damaged salad leaves as a source of nutrients.

Furthermore, human pathogens belonging to the genus *Clostridium* are able to survive the waste water treatment process [60]. In our experiments, the taxon *Clostridium* (sensu stricto) was enriched in samples containing sludge at later timepoints and was less abundant at early timepoints. It was low abundant in samples without CSS. This underlines the potential risk of contamination in soil caused by CSS. Besides pathogenic bacteria, sewage sludge and other organic fertilizers are abundant in antibiotic resistance genes [53,61,62]. Sewage sludge, as a by-product in WWTPs, is extensively used worldwide as an organic soil amendment and could pose a viable strategy for nutrient recovery from municipal waste [14]. Although some mandatory limits for heavy metals in sewage sludge (Cd: 20–40 mg/kg; Cu: 1,000–1,750 mg/kg; Hg: 16–25 mg/kg; Ni: 300–400 mg/kg; Pb: 750–1,200 mg/kg and Zn: 2,500–4,000 mg/kg) have been proposed by the European Commission for sewage sludge soil application [13], this could escalate the antibiotic resistance crisis [63].

## 5. Conclusions

Our results revealed that within the soil prokaryotic community, Proteobacteria, Actinobacteria, Acidobacteria, Bacteroidetes, Chloroflexi and Verrucomicrobia increased in relative abundance during plant growth, while a decrease was observed for Firmicutes, Acidobacteria, Crenarchaeota and Nitrospirae. The addition of CSS boosted the relative abundances of Firmicutes and Chloroflexi and decreased the abundance of Acidobacteria. Importantly, our results suggest that if *Salmonella* is applied to the soil together with CSS, its persistence is longer, compared to soil without composted sewage sludge, or compared to when it was introduced with water to fertilized soil at the time of planting. Finally, when assessing the colonization rate of the *B. rapa* phyllosphere on soil inoculated with *Salmonella*, our results revealed that between 1.9% and 3.6% of plants were colonized with *Salmonella*. Although we have seen different persistence dynamics for both *Salmonella* strains in composted sewage sludge amended soil, this had no impact on the plant colonization pattern. The plant colonization rate however, seemed higher when *Salmonella* was introduced on planting day. Taking together, even though the use of CSS is an interesting option, safety measures should be applied in order to avoid crop contamination.

## Figures and Tables

**Table 1:** The soil microbial community on phylum level is influenced by time and addition of sludge compost. However, it is not influenced by the presence of *Salmonella.* Relative abundance of phyla is expressed as a percentage of the total bacterial population. Results were evaluated using Student’s *t*-test with Benjamini–Hochberg adjusted *p* values, with *p* ≤ 0.05 considered significant (bold). The results are expressed as mean ± SD from four biological replicates (*n* = 43).

	Time			Sludge			*Salmonella*		
	Planting Day *n* = 16	35 dpi *n* = 23	*p*-Value	Control *n* = 23	Sludge *n* = 20	*p*-Value	Control *n* = 19	14028s *n* = 24	*p*-Value
Proteobacteria	20.1 ± 1.5	30.5 ± 3.8	*˂0.001*	26.3 ± 6.2	25.0 ± 5.9	0.946	24.1 ± 6.0	26.9 ± 5.9	0.731
Actinobacteria	18.8 ± 1.3	20.3 ± 1.8	*0.003*	19.6 ± 2.1	19.7 ± 1.2	0.918	19.6 ± 2.0	19.7 ± 1.5	0.606
Firmicutes	20.7 ± 2.7	13.2 ± 3.3	*˂0.001*	15.6 ± 4.0	17.9 ± 5.4	*˂0.001*	17.9 ± 5.4	15.8 ± 4.2	0.924
Acidobacteria	16.3 ± 4.0	14.8 ± 1.0	*˂0.001*	16.1 ± 1.5	14.8 ± 1.0	*˂0.001*	15.6 ± 1.3	15.4 ± 1.6	0.909
Crenarchaeota	10.9 ± 1.2	6.2 ± 1.4	*˂0.001*	8.6 ± 2.9	8.2 ± 2.5	0.869	8.9 ± 2.8	8.0 ± 2.6	0.452
Bacteroidetes	5.8 ± 0.7	7.6 ± 1.3	*˂0.001*	6.7 ± 1.1	6.9 ± 1.7	0.848	6.5 ± 1.0	7.0 ± 1.7	0.919
Gemmatimonadetes	2.3 ± 0.3	2.3 ± 0.2	0.507	2.3 ± 0.2	2.3 ± 0.3	1.000	2.3 ± 0.3	2.4 ± 0.2	0.522
Chloroflexi	1.6 ± 0.3	1.8 ± 0.3	*0.035*	1.6 ± 0.2	1.9 ± 0.3	*˂0.001*	1.7 ± 0.3	1.7 ± 0.3	0.517
Nitrospirae	1.3 ± 0.1	0.9 ± 0.2	*˂0.001*	1.1 ± 0.3	1.1 ± 0.2	0.961	1.2 ± 0.2	1.1 ± 0.3	0.566
TM7	1.0 ± 0.2	0.9 ± 0.3	0.729	1.0 ± 0.2	0.9 ± 0.3	1.000	1.0 ± 0.2	0.9 ± 0.2	0.481
Verrucomicrobia	0.8 ± 0.1	1.1 ± 0.1	*˂0.001*	0.9 ± 0.2	1.0 ± 0.2	0.888	0.9 ± 0.2	1.0 ± 0.1	0.616

**Table 2:** *Salmonella enterica* colonized plants expressed as a percentage of the tested population. Phyllosphere of *B. rapa* plants was tested for the presence of *Salmonella* using the Buffered Peptone Water enrichment technique followed by *Salmonella*-specific cultivation in the Rappaport–Vassiliadis broth. Both enrichments were plated on XLD plates supplemented with rifampicin to verify *Salmonella’s* presence. We tested *n* = 678 plants. Two plants were pooled together in order to reduce the number of samples. This results in the min. (%) and max. (%) number of colonized plants. Significant differences were calculated using one-way ANOVA and are highlighted in bold (*p* ≤ 0.05).

	Soil		Serovar		Scenario		Days after Planting				Total
	DS soil	DS soil + CSS	14028s	Senftenberg	Fresh	Stored	7	21	35	56	
Min (%)	1.9	1.9	1.9	1.9	2.8	0.9	1.9	5.6	0.0	0.0	1.9
Max (%)	3.8	3.8	3.8	3.8	5.6	1.9	3.8	11.3	0.0	0.0	3.8
*p*-value	1.000		1.000		0.066		**0.001**				

## Supplementary Materials

The following are available online at https://www.mdpi.com/2076-2607/8/7/1020/s1, Table S1: Pearson’s correlations between phyla. Table S2: Taxonomic groups with relative abundance different between treatments.
