# Supplementary material for: Composted Sewage Sludge Influences the Microbiome and Persistence of Human Pathogens in Soil
Source: Microorganisms. 2020 Jul 9;8(7):1020. doi: 10.3390/microorganisms8071020 (PMC7409118; doi:10.3390/microorganisms8071020)
Supplement: Supplementary file 1 [file microorganisms-08-01020-s001.zip › Supplementary Tab S1.docx]

## Supplementary Information

**Supplementary Table S1**. Pearson’s correlations between phyla

Statistically significant correlations (*p* ≤0.05) are highlighted in red.

|  | Proteobacteria | Actinobacteria | Firmicutes | Acidobacteria | Crenarchaeota | Bacteroidetes | Gemmatimonadetes | Chloroflexi | Nitrospira | TM7 |
| --- | --- | --- | --- | --- | --- | --- | --- | --- | --- | --- |
| Proteobacteria | 1,00 |  |  |  |  |  |  |  |  |  |
| Actinobacteria | 0,22 | 1,00 |  |  |  |  |  |  |  |  |
| Firmicutes | *-0,92* | -0,21 | 1,00 |  |  |  |  |  |  |  |
| Acidobacteria | *-0,50* | *-0,34* | 0,27 | 1,00 |  |  |  |  |  |  |
| Crenarchaeota | *-0,86* | *-0,42* | *0,68* | *0,55* | 1,00 |  |  |  |  |  |
| Bacteroidetes | *0,59* | -0,01 | *-0,57* | *-0,59* | *-0,48* | 1,00 |  |  |  |  |
| Gemmatimonadetes | -0,09 | -0,05 | 0,00 | *0,31* | 0,13 | *-0,35* | 1,00 |  |  |  |
| Chloroflexi | 0,22 | -0,07 | -0,11 | *-0,34* | *-0,40* | 0,21 | -0,18 | 1,00 |  |  |
| Nitrospira | *-0,76* | -0,19 | *0,70* | *0,43* | *0,63* | *-0,58* | 0,09 | -0,07 | 1,00 |  |
| TM7 | -0,06 | -0,05 | 0,01 | 0,02 | 0,05 | 0,10 | -0,15 | -0,04 | 0,24 | 1,00 |
| Verrucomicrobia | *0,65* | -0,07 | *-0,67* | -0,24 | *-0,53* | *0,49* | 0,02 | 0,27 | *-0,68* | -0,25 |
